# Serum levels of AgRP protein in patients with schizophrenia on clozapine monotherapy

**DOI:** 10.1007/s11011-014-9592-6

**Published:** 2014-07-19

**Authors:** Adam Wysokiński, Jakub Kaźmierski, Iwona Kłoszewska

**Affiliations:** Department of Old Age Psychiatry and Psychotic Disorders, Medical University of Lodz, Czechosłowacka 8/10, 92-216 Łódź, Poland

**Keywords:** Schizophrenia, Clozapine, AgRP, Metabolic syndrome

## Abstract

Aim: Agouti-related peptide (AgRP) is one of the hypothalamic hormones that works by increasing appetite and decreasing metabolism, thus leading to weight gain. The aim of the study was to find out if AgRP level in subjects with schizophrenia on clozapine monotherapy is higher compared with healthy controls. Methodology: We determined fasting serum AgRP levels in 24 subjects with schizophrenia on clozapine monotherapy and 24 healthy, age- and sex-matched controls. Biochemical and anthropometric measurements were combined with body composition analysis. Results: There was no difference for AgRP levels between patients taking clozapine and control group (15.00±8.65 vs. 15.33±6.82 pg/mL, *p* =0.37). We found negative correlations between AgRP levels and total body fat (*r* =−0.34 and −0.48 in the whole study group and clozapine group, respectively) and positive correlations with lean body mass (*r* =0.38 and 0.49 in the whole study group and clozapine group, respectively), body water (*r* =0.34 and 0.49 in the whole study group and clozapine group, respectively) and basal metabolic rate (*r* =0.42 both in the clozapine and control groups). There were no correlations with age, height, weight, body mass index, fat mass index, abdominal, waist or hip circumferences, waist-hip ratio, blood pressure, total cholesterol, HDL, LDL, triglycerides, uric acid, glucose, insulin, clozapine dose or treatment duration, duration of treatment with antipsychotics and markers for insulin resistance. Conclusion: We cannot conclude that treatment with clozapine is associated with increased level of AgRP. We did not find previously described differences in AgRP levels between obese and non-obese subjects or associations between AgRP and various metabolic parameters.

## Introduction

Clozapine remains an ultimate option for patients with treatment resistant schizophrenia (Kane 2012). Efficacy of clozapine against positive symptoms of schizophrenia was confirmed in numerous studies and meta-analyses (Chakos et al. 2001), including analysis published by the Cochrane Library (Asenjo Lobos et al. 2010). However, treatment with clozapine is associated with very severe metabolic side-effects (Newcomer 2005). Clozapine-induced weight gain is very common (Wetterling 2001), and so is impaired fasting plasma glucose levels. In patients with schizophrenia weight gain is associated with impaired physical functioning and negative body appraisal (Bachmann et al. 2012), both of which affect quality of life. While treatment with clozapine may reduce mortality by reducing suicide rate, mortality due to clozapine-associated weight gain will diminish reduction in the suicide rate almost entirely over 10 years by the increased mortality associated with weight gain of 10 kg (Fontaine et al. 2001). Also, obesity is linked with numerous secondary health problems (pressure overload on lungs, joints and bones) and is an important risk factor for life-threatening diseases, such as cardiovascular disease, type 2 diabetes and certain cancers.

Several mechanisms are considered as taking role in clozapine-induced weight gain, including antagonism at histamine H1 receptors (Kroeze et al. 2003) and serotonin 5-HT1B and 5-HT2C receptors. These mechanisms may also include 5-HT2A receptors (Rasmussen et al. 2014). Various receptors, including aforementioned 5-HT1B and 5-HT1C, regulate activity of the hypothalamic nuclei (particularly the arcuate nucleus (ARC), which plays the key role in appetite regulation). Activity of ARC is also regulated by several hormones of anorexigenic properties: leptin, pancreatic polypeptide (PP), cholecystokinin (CCK), glucagon-like peptide-1 (GLP-1), oxyntomodulin (OXM), peptide YY (PYY) and the first discovered orexigenic substance - ghrelin (Druce et al. 2004).

Agouti-related peptide (AgRP) is one of the hypothalamic hormones that works by increasing appetite and decreasing metabolism and energy expenditure, thus leading to weight gain. AgRP is a paracrine signaling molecule made up of 132 amino acids. It was identified in 1997 based on its sequence similarity with Agouti signaling peptide (ASIP), a protein synthesized in the skin that controls coat color. Compared with ASIP, AgRP is physiologically expressed in the hypothalamus (Shutter et al. 1997). AgRP is also expressed in the adrenal gland, testes, kidneys, and lungs. AgRP induces obesity by antagonism of the melanocortin receptors (MC3-R and MC4-R, subtypes implicated in weight regulation, via metabolism and appetite control) (Ollmann et al. 1997). In the brain AgRP is produced in ARC by the AgRP/NPY neurons. These neurons make peptides that potently stimulate food intake not only by increasing neuropeptide Y (NPY) signaling, but also by reducing melanocortin signaling via the release of AgRP (Morton and Schwartz 2001). Activity of the AgRP/NPY neurons is modulated by leptin, released from the adipose tissue, and insulin. The adiposity signals (insulin and leptin) are secreted in proportion to body fat content and act in the hypothalamus to inhibit anabolic and stimulate catabolic, effector pathways (Schwartz et al. 2000). Compared with NPY, AgRP has long-lasting orexigenic properties. The increase of food intake following a single central administration of AgRP is sustained for up to a week (Hagan et al. 2000), while the response to NPY is sustained over hours, rather than days.

This study was undertaken with the purpose to determine if patients with schizophrenia on clozapine monotherapy have higher fasting levels of AgRP compared with healthy control. In order to provide more accurate measurements, biochemical and anthropometric measurements were combined with body composition determined using bioelectric impedance analysis (BIA), which provides accurate measurements of body fat, lean mass and body water (Bosy-Westphal et al. 2008). To the best of our knowledge, this is the first study to investigate such combination of these parameters in subjects with schizophrenia treated with clozapine.

## Material and methods

Data for 24 European Caucasian adult hospitalized patients with paranoid schizophrenia (295.30 according to DSM-IV, F20.0 according to ICD-10) was included into the study. These subjects were on clozapine monotherapy for at least two months prior the assessments with a minimum dose of 100 mg/day. Most patients was in stable phase of the disease (i.e. no acute psychosis). Control group was 24 healthy subjects and was gender- and age-matched with patients in the clozapine group. Health status of the control subjects was determined on the basis of basic physical examination, including vital signs and an interview. Patients/controls with substance abuse/dependence were excluded from the study. All patients and volunteers included in the study expressed their written informed consent for participation in this study. The study protocol was approved by the local Bioethics Committee. There was no financial involvement from the industry.

The blood samples for the chemistry panel were collected between 7 am and 8 am, after ensuring at least 8 h of overnight fasting. The samples were immediately transferred to the central laboratory where they were analyzed. Glucose, lipids, calcium and uric acid levels were measured using a Dirui CS-400 analyzer (Dirui, China). Homocysteine chemiluminescence assessments were performed using an Immulite 2,000 analyzer (Siemens, Germany), insulin immunochemistry assessments were performed using a Cobas E411 analyzer (Roche Diagnostics, Switzerland) and albumin levels were assessed using a Cobas Integra 800 analyzer (Roche Diagnostics, Switzerland). Levels of AgRP were measured in blood serum using ELISA (enzyme-linked immunosorbent assay) method. Prior to assays, serum samples were stored at −80 °C for up to 6 months. ELISA assays were performed using commercial kits (intra-assay: CV <10 %, inter-assay: CV <12 %) manufactured by RayBiotech (USA), according to protocol provided by its manufacturer (all samples were 2-fold diluted).

Metabolic syndrome and abdominal obesity were defined according to International Diabetes Foundation (IDF) criteria (Alberti et al. 2006). Impaired fasting glucose was defined as fasting plasma glucose ≥100 mg/dL. BMI <25 kg/m^2^, 25–30 kg/m^2^ and ≥30 kg/m^2^ were defined as normal weight, overweight and obesity, respectively. Raised triglycerides (TGA) level ≥150 mg/dL and/or total cholesterol (TC) ≥200 mg/dL and/or reduced HDL cholesterol level <40 mg/dL for men and <50 mg/dL for women and/or raised LDL cholesterol level ≥135 mg/dL were interpreted as dyslipidemia. Corrected calcium was calculated using the formula: corrected calcium [mg/dL] = measured total calcium [mg/dL] +0.8 (4.0− serum albumin [g/dL]). Insulin resistance was estimated from fasting glucose and insulin results by homeostasis model assessment and QUICKI index, using the formula: HOMA1-IR = (fasting plasma glucose [mg/dL] × insulin [mU/L])/405. HOMA2-IR index was calculated using a calculator downloaded from http://www.dtu.ox.ac.uk. QUICKI index (lower numbers reflect greater insulin resistance) was calculated using the formula: 1/(log (fasting insulin [mU/L]) + log (fasting plasma glucose [mg/dL])). Insulin resistance was defined as HOMA1-IR >2.0.

Height was measured with a wall-mounted height measure to the nearest 0.5 cm. Weight was measured with a spring balance that was kept on a firm horizontal surface. Subjects wore light clothing, stood upright without shoes and weight was recorded to the nearest 0.5 kg. Body mass index (BMI) was calculated as body weight in kilogram divided by the height in meter squared (kg/m^2^). Waist, abdominal and hip circumference was measured using a non-stretchable fiber measuring tape. Waist-to-hip ratio (WHR) was calculated as waist circumference divided by hip circumference. WHR cut-off points were defined according to WHO recommendations (0.85 for women and 0.9 for men). Fat mass index (FMI) was calculated as total body fat in kilogram divided by the height in meter squared (kg/m^2^). Excess body fat according to FMI classification ranges were defined as FMI >6 for men and FMI >9 for women (Kelly et al. 2009).

Body composition was measured using a Maltron BF-906 Body Fat Analyzer (Maltron, UK), single frequency bioelectrical impedance analyzer to determine resistance and reactance at 50 Hz. Standard operating conditions were observed by a trained operator including preparation of the participant, electrode placement and operation. The measurement using BIA was taken immediately prior to anthropometry measurements with participants lying supine, in a rested state.

Statistical procedures were performed with STATA 13.1 (StataCorp, USA). Simple descriptive statistics (means, standard deviations, 95 % confidence interval [CI]) were generated for all continuous variables. For discrete variables number of patients and percentages are given. Normality of distribution was tested with Shapiro-Wilk test. Skewed variables were transformed to follow normal distribution using log, square root, inverse square root or square transformation. The method of transformation was chosen empirically for best normality. Means, standard deviations, and confidence intervals are reported for non-transformed variables, results of tests are reported for transformed or non-transformed variables. If transformation resulted in normal distribution, two-tailed *t*-test was used to assess inter-group differences, otherwise variables were analyzed using Mann–Whitney *U* test. The difference between proportions was analyzed by Fisher’s exact test. Associations were tested by Pearson’s (for variables with normal distribution) or Spearman's (for other variables) correlation coefficients. The significant level was set at *p* <0.05.

## Results

For group of patients treated with clozapine the mean age was 38.8±12.6 and 39.9±12.3 for the control group (*p* =0.62). In both groups there were 12 men and 12 women. In the clozapine group 12 subjects smoked cigarettes and 8 in the control group (*p* =0.38). The mean duration of monotherapy with clozapine was 60.5±79.4 [95 % CI: 27.0–94.1] months and mean clozapine dose was 341.1±148.6 [95 % CI: 278.4–403.8] mg/day. Detailed results for anthropometric measurements and laboratory tests are shown in Table 1.

**Table 1:** Results of anthropometric measurements and laboratory tests

	clozapine *n* =24	control *n* =24	
Weight [kg]	78.1±14.2 (72.1–84.1)	72.6±15.1 (66.2–78.9)	*p* =0.08 *t* =1.38
BMI [kg/m^2^]	27.1±3.6 (25.5–28.6)	24.8±3.5 (23.3–26.2)	*p* =0.01 *t* =2.25
FMI [kg/m^2^]	8.9±3.1 (7.6–10.2)	7.7±3.0 (6.4–9.0)	*p* =0.09 *t* =1.38
Abdominal circumference [cm]	96.5±9.4 (92.5–100.4)	85.5±11.6 (80.6–90.3)	*p* <0.001 *z* =3.58
Waist circumference [cm]	91.1±12.1 (86.0–96.2)	82.4±10.6 (77.8–86.8)	*p* =0.005 *z* =2.66
Hip circumference [cm]	99.0±8.4 (95.5–102.5)	95.9±7.1 (92.9–98.9)	*p* =0.08 *t* =1.39
WHR	0.92±0.08 (0.88–0.95)	0.86±0.08 (0.82–0.89)	*p* =0.005 *z* =2.66
SBP [mm Hg]	121.7±13.6 (115.9–127.4)	136.7±17.9 (129.1–144.2)	*p* =0.001 *z* =−3.26
DBP [mm Hg]	81.2±8.5 (77.6–84.8)	82.8±12.1 (77.6–87.9)	*p* =0.30 *t* =−0.50
Uric acid [mg/dL]	4.5±1.4 (3.9–5.1)	4.3±1.3 (3.7–4.8)	*p* =0.28 *t* =0.57
Homocysteine [μmol/L]	14.5 ± 4.4 (12.6–16.4)	13.6 ± 5.0 (11.5–15.7)	*p* = 0.25 *t* = 0.67
TC [mg/dL]	194.2 ± 53.2 (171.7–216.6)	216.6 ± 65.3 (189.0–244.1)	*p* = 0.06 *t* = 1.54
HDL [mg/dL]	43.5 ± 12.6 (38.2–48.8)	55.1 ± 14.3 (49.1–61.1)	*p* = 0.002 *t* =−2.97
LDL [mg/dL]	122.6 ± 41.9 (104.8–140.3)	128.3 ± 39.7 (111.5–145.0)	*p* = 0.29 *t* =−0.54
TGA [mg/dL]	140.3 ± 120.4 (89.4–191.1)	104.3 ± 81.4 (69.9–138.6)	*p* = 0.07 *t* = 1.50
FPG [mg/dL]	103.5 ± 31.7 (90.1–116.8)	87.8 ± 11.7 (82.8–92.7)	*p* = 0.03 *t* =−1.88
Insulin [μU/mL]	11.8 ± 8.2 (8.3-15.3)	7.7 ± 3.2 (6.3-9.1)	*p* = 0.02 *t* =−2.08
HOMA1-IR	3.3 ± 3.4 (1.8–4.7)	1.7 ± 0.8 (1.3–2.0)	*p* = 0.009 *t* =−2.46
HOMA2-IR	1.6 ± 1.1 (1.1–2.1)	1.0 ± 0.4 (0.8–1.2)	*p* = 0.01 *t* =−2.26
QUICKI	0.15 ± 0.01 (0.14–0.15)	0.16 ± 0.01 (0.15–0.16)	*p* = 0.007 *t* =−2.52
Corrected calcium [mg/dL]	8.6 ± 0.9 (8.2–9.0)	8.7 ± 0.7 (8.4–9.0)	*p* = 0.37 *t* =−0.33

Data given as: mean ± standard deviation (95 % CI)

BMI = body mass index; FMI = fat mass index; WHR = waist-to-hip ratio; SBP = systolic blood pressure; DBP = diastolic blood pressure; TC = total cholesterol; HDL = high density lipoproteins; LDL = low density lipoproteins; TGA = triglycerides; FPG = fasting plasma glucose; HOMA1-IR = homoeostasis model assessment of insulin resistance 1; HOMA2-IR = homoeostasis model assessment of insulin resistance 2; QUICKI = quantitative insulin sensitivity check index

We have found no inter-group differences for body composition analysis. Detailed results for BIA analysis are shown in Table 2. Lean body mass was higher in men in the whole study sample (60.1±6.4 [95 % CI: 57.4-62.8] vs. 43.8±5.4 [95 % CI: 41.5–46.1] kg, *t* =9.56, p <0.001) and in the clozapine group (59.6±5.7 [95 % CI: 56.0–63.3] vs. 45.3±7.0 [95 % CI: 40.9-49.8] kg, *t* =5.48, p <0.001). Similarly, basal metabolic rate was higher in men in the whole study sample (1,707.7±182.3 [95 % CI: 1,630.7–1,784.6] vs. 1,337.3±138.4 [95 % CI: 1,278.8-1,395.7] kcal/day, *t* =7.92, *p* <0.001) and in the clozapine group (1,701.2±138.2 [95 % CI: 1,613.4–1,789.0] vs. 1,362.7±173.0 [95 % CI: 1,252.7–1,472.5] kcal/day, *t* =5.29, p <0.001).

**Table 2:** Results of body composition analysis

	clozapine *n* = 24	control *n* = 24	
Total body fat [kg]	25.6 ± 8.8 (21.8–29.2)	22.4 ± 8.7 (18.7–26.1)	*p* = 0.10 *t* = 1.25
Total body fat [%]	32.6 ± 8.4 (29.1–36.2)	28.9 ± 7.1 (25.8–31.8)	*p* = 0.06 *t* = 1.67
Lean body mass [kg]	52.5 ± 9.6 (48.4–56.5)	51.4 ± 10.8 (46.8–55.9)	*p* = 0.36 *t* = 0.36
Lean body weight [%]	67.6 ± 8.1 (64.2–71.0)	71.1 ± 7.1 (68.1–74.1)	*p* = 0.07 *t* =−1.61
Total body water [L]	38.4 ± 7.0 (35.4–41.3)	37.7 ± 7.9 (34.3–40.9)	p = 0.36 t = 0.35
Total body water [%]	49.9 ± 5.4 (47.6–52.1)	52.1 ± 5.2 (49.8–54.2)	*p* = 0.09 *t* = −1.42
Basal metabolic rate [kcal/day]	1,532.0 ± 230.9 (1,434.4–1,629.4)	1,513.0 ± 265.4 (1,400.9–1,625.1)	*p* = 0.39 *t* = 0.26

Data given as: mean ± standard deviation (95 % CI)

**Fig. 1:**
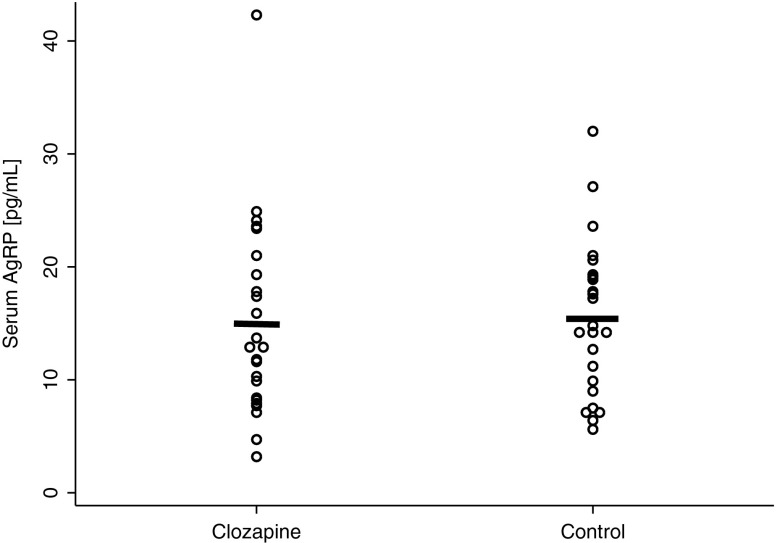
Mean fasting AgRP serum levels [pg/mL] in subjects on clozapine and in the control group (*p* =0.37; horizontal bars indicate means).

There were no significant difference for fasting serum levels of AgRP between the clozapine group and the control group (15.00±8.65 [95 % CI: 11.34–18.65] vs. 15.33±6.82 [95 % CI: 12.45–18.22] pg/mL, *t* =−0.32, *p* =0.37), see Fig. 1. Women had significantly lower levels of AgRP in the whole study group (12.53±5.65 [95 % CI: 10.14–14.92] vs. 17.80±8.66 [95 % CI: 14.14–21.45] pg/mL, *t* =2.47, *p* =0.009) and in the control group (12.90±5.77 [95 % CI: 9.22–16.57] vs. 17.77±7.14 [95 % CI: 13.23–22.31] pg/mL, *t* =1.82, *p* =0.04), but the difference was not significant for the clozapine group (12.17±5.75 [95 % CI: 8.51–15.83] vs. 17.82±10.28 [95 % CI: 11.28-24.36] pg/mL, *t* =1.64, *p* =0.06).

For the whole study group significant correlations of AgRP levels were found for total body fat [%] (*r* =−0.34, *p* =0.02), lean body mass [kg] (*r* =0.38, *p* =0.006), lean body mass [%] (*r* =0.34, *p* =0.02), body water [l] (*r* =0.38, *p* =0.006), body water [%] (*r* =0.34, *p* =0.02) and homocysteine levels (*r* =0.29, *p* =0.04). For the clozapine group significant correlations of AgRP levels were found for total body fat [%] (*r* =−0.48, *p* =0.02), basal metabolic rate (*r* =0.42, *p* =0.04), lean body mass [%] (*r* =0.49, *p* =0.01), body water [%] (*r* =0.49, *p* =0.01). For the control group significant correlations of AgRP levels were found only for basal metabolic rate (*r* =0.42, *p* =0.04).

In the clozapine group there were no significant differences for AgRP levels between subjects with or without excess body fat (based on FMI value) (*p* =0.59), IDF-defined metabolic syndrome (*p* =0.33), smokers and non-smokers (*p* =0.15), subjects with BMI <25 kg/m^2^ and with BMI ≥25 kg/m^2^ (*p* =0.09), subjects with and without impaired fasting glucose (*p* =0.16), subjects with and without abdominal obesity (*p* =0.11), subjects with and without dyslipidemia (*p* =0.09), and subjects with and without HOMA-1IR defined insulin resistance (*p* =0.07).

In the control group there were no significant differences for AgRP levels between subjects with or without excess body fat (based on FMI value) (*p* = 0.20), IDF-defined metabolic syndrome (*p* = 0.44), smokers and non-smokers (*p* = 0.35), subjects with BMI <25 kg/m^2^ and with BMI ≥25 kg/m^2^ (*p* =0.12), subjects with and without impaired fasting glucose (*p* =0.36), subjects with and without abdominal obesity (*p* =0.10), subjects with and without dyslipidemia (*p* =0.37), and subjects with and without HOMA-1IR defined insulin resistance (*p* =0.13).

In the whole study group there was no significant differences for AgRP levels between subjects with or without excess body fat (based on FMI value) (*p* =0.30), IDF-defined metabolic syndrome (*p* =0.43), smokers and non-smokers (*p* =0.29), subjects with BMI <25 kg/m^2^ and with BMI ≥25 kg/m^2^ (*p* =0.39), subjects with and without impaired fasting glucose (*p* =0.29), subjects with and without dyslipidemia (*p* =0.11), and subjects with and without HOMA-1IR defined insulin resistance (*p* =0.33). There was a significant difference for AgRP levels between subjects with or without abdominal obesity (13.46±6.50 [95 % CI: 9.09–17.83] vs. 16.92±6.93 [95 % CI: 12.73–21.11] pg/mL, *t* =1.80, *p* =0.04).

## Discussion

The main objective of the present study was to find out whether there is a significant difference in fasting serum levels of AgRP peptide between patients with schizophrenia on clozapine monotherapy and age- and sex-matched healthy controls. We have found the difference was not significant (clozapine: 15.00±8.65, control: 15.33±6.82 pg/mL, *p* =0.37). We do not know pre-treatment values, so we cannot determine whether and how this parameter changed during therapy with clozapine. To our knowledge, no data are available on the effect of clozapine on blood AgRP concentrations in humans. Limited data are available for olanzapine, which has clinical and pharmacological properties similar to clozapine. Results of these studies show that treatment with olanzapine does not affect AgRP levels (Basoglu et al. 2010; Ehrlich et al. 2012). However, in animal study it was demonstrated that administration of olanzapine or clozapine upregulates NPY and AgRP and downregulates proopiomelanocortin in the arcuate nucleus of the hypothalamus (Ferno et al. 2011). We do not know studies demonstrating correlation between blood levels of AgRP and levels in the hypothalamus, but since it was showed recently that AgRP neurons are unique among hypothalamic neurons by being the predominant neuronal subtype situated outside the blood–brain barrier (Olofsson et al. 2013), we may assume that changes in hypothalamic expression are reflected in changes in blood levels.

The finding that women had significantly lower levels of AgRP in the whole study group (12.53±5.65 vs. 17.80±8.66 pg/mL, *p* =0.009) and in the control group (12.90±5.77 vs. 17.77±7.14 pg/mL, *p* =0.04), but not in the clozapine group (12.17±5.75 vs. 17.82±10.28 pg/mL, *p* =0.057), might indicate that the testes are important source of AgRP, as shown previously (Shutter et al. 1997). However, several more recent studies showed no difference between men and (Gavrila et al. 2005; Katsuki et al. 2001), therefore it may indicate that plasma AgRP levels reflect central AgRP concentrations.

Levels of AgRP might also not be directly related to treatment with clozapine. It was previously reported that total level of AgRP is higher in obese subjects (Katsuki et al. 2001), although in another study it has been demonstrated that AgRP levels were lower in normal weight group compared with women with anorexia, probably due to increased leptin levels (Moriya et al. 2006). We did not measure levels of leptin or acylated ghrelin, but we found in the same group of patients that there were no differences for desacyl ghrelin levels between patients taking clozapine and control group (Wysokiński et al. 2014). While there was a significant difference in BMI values between the clozapine group and the control group, we found no differences between both groups for FMI values. Compared with BMI, FMI is a better indicator of central obesity since it is much more sensitive to body fat content (Kelly et al. 2009), while BMI may be increased by muscle mass. Moreover, there were no differences for any of the BIA results between both groups. These indicate that there was a substantial similarity between both groups in terms of body fat content, which is important considering that AgRP levels may affect or result more from the amount of adipose tissue than from body weight per se. On the other hand, we have found negative correlations between AgRP levels and total body fat (*r* =−0.34 and −0.48 in the whole study group and clozapine group, respectively). These may indicate the effect of leptin on the expression of AgRP, but require further studies. Additionally, there were positive correlations with lean body mass (*r* =0.38 and 0.49 in the whole study group and clozapine group, respectively), body water (which amount is negatively correlated with the amount of body fat: *r* =−0.96, *p* < 0.001) (*r* =0.34 and 0.49 in the whole study group and clozapine group, respectively) and basal metabolic rate (*r* =0.42 both in the clozapine group and control group), also being in line with the above-mentioned mechanism.

In animal studies it has been found that high-fat diet leads to decreased expression of AgRP in the hypothalamus, probably in the mechanism moderated by leptin (Staszkiewicz et al. 2007). We have no detailed data on diet patterns of our study subjects, but reports are consistent that patients with schizophrenia consume higher amounts of high-fat foods compared with healthy population (Dipasquale et al. 2013). This might be one of the reasons why we have found no differences between our study groups. Also, level of physical activity (usually lower in hospitalized patients) might affect mechanisms regulating AgRP levels.

Finally, another important factor is the effect of treatment mediated by other anabolic or catabolic neuropeptides. Here, two most important peptides are leptin (which decreases AgRP levels) and ghrelin (which has opposite effect) and have also been studied most extensively (Sentissi et al. 2008). Since both leptin and ghrelin (Andrews 2011) may regulate activity of AgRP/NPY neurons in the arcuate nucleus, effects of antipsychotics on these neuropeptides must be taken into consideration. Therefore, a study focused on interactions within this regulatory system would be very important for better understanding of this problem.

Based on our results, we cannot conclude that treatment with clozapine affects levels of AgRP. However, the low number of study subjects limited the probability of finding inter-group differences due to lack of statistical power. Due to the cross-sectional study design causal relationships cannot be established and the effect of previous antipsychotic treatment cannot be excluded. Dual-energy X-ray absorptiometry (DXA) should be used to measure body composition and adipose tissue mass more accurately. Due to complex structure of interactions between anabolic and catabolic neuropeptides, a longitudinal study comparing these interactions are crucial for understanding mechanisms of treatment-induced weight gain.
